# Digital Microfluidic Platform to Maximize Diagnostic Tests with Low Sample Volumes from Newborns and Pediatric Patients

**DOI:** 10.3390/diagnostics10010021

**Published:** 2020-01-01

**Authors:** Rama S. Sista, Rainer Ng, Miriam Nuffer, Michael Basmajian, Jacob Coyne, Jennifer Elderbroom, Daniel Hull, Kathryn Kay, Maithri Krishnamurthy, Christopher Roberts, Daniel Wu, Adam D. Kennedy, Rajendra Singh, Vijay Srinivasan, Vamsee K. Pamula

**Affiliations:** Baebies, Inc., PO Box 14403, Durham, NC 27709, USA; rsista@baebies.com (R.S.S.); rng@baebies.com (R.N.); mnuffer@baebies.com (M.N.); mbasmajian@baebies.com (M.B.); jcoyne@baebies.com (J.C.); jelderbroom@baebies.com (J.E.); dhull@baebies.com (D.H.); kkay@baebies.com (K.K.); mkrishnamurthy@baebies.com (M.K.); croberts@baebies.com (C.R.); dwu@baebies.com (D.W.); akennedy@baebies.com (A.D.K.); rsingh@baebies.com (R.S.); vsrinivasan@baebies.com (V.S.)

**Keywords:** pediatrics, laboratory, digital microfluidics, electrowetting, nanoliter, neonatal intensive care unit (NICU), bilirubin, glucose-6-phosphate dehydrogenase, albumin, thyroid, insulin, creatinine, cytomegalovirus

## Abstract

“Children are not tiny adults” is an adage commonly used in pediatrics to emphasize the fact that children often have different physiological responses to sickness and trauma compared to adults. However, despite widespread acceptance of this concept, diagnostic blood testing is an excellent example of clinical care that is not yet customized to the needs of children, especially newborns. Cumulative blood loss resulting from clinical testing does not typically impact critically ill adult patients, but can quickly escalate in children, leading to iatrogenic anemia and related comorbidities. Moreover, the tests prioritized for rapid, near-patient testing in adults are not always the most clinically relevant tests for children or newborns. This report describes the development of a digital microfluidic testing platform and associated clinical assays purposely curated to address current shortcomings in pediatric laboratory testing by using microliter volumes (<50 µL) of samples. The automated platform consists of a small instrument and single-use cartridges, which contain all reagents necessary to prepare the sample and perform the assay. Electrowetting technology is used to precisely manipulate nanoliter-sized droplets of samples and reagents inside the cartridge. To date, we have automated three disparate types of assays (biochemical assays, immunoassays, and molecular assays) on the platform and have developed over two dozen unique tests, each with important clinical application to newborns and pediatric patients. Cell lysis, plasma preparation, magnetic bead washing, thermocycling, incubation, and many other essential functions were all performed on the cartridge without any user intervention. The resulting assays demonstrate performance comparable to standard clinical laboratory assays and are economical due to the reduced hands-on effort required for each assay and lower overall reagent consumption. These capabilities allow a wide range of assays to be run simultaneously on the same cartridge using significantly reduced sample volumes with results in minutes.

## 1. Introduction

Clinical laboratory tests are an invaluable component of medical care that aid in both the diagnosis of disease and the evaluation of response to treatment. Unfortunately, for neonatal and pediatric patients undergoing intense medical interventions, significant challenges may arise from the diagnostic blood sampling process. Cumulative blood loss from diagnostic testing can escalate quickly in critically ill patients resulting in iatrogenic anemia and need for transfusions, which are associated with higher risk of infection, donor erythrocyte injury, and other cardiac, vascular, and hemorrhagic complications [1,2,3,4]. In a multi-center study of 30 pediatric intensive care units (PICUs), 74% of critically ill children were anemic and the majority of daily blood loss was attributed directly to blood draws for diagnostic testing [5]. 

The dangers of iatrogenic anemia have gained particular attention for newborns in the neonatal intensive care unit (NICU) setting [6], as the average total blood volume for a term newborn is only 240 mL and can be as little as 60 mL in extremely low birth weight (ELBW) neonates [6]. Over several days of testing, the relatively large volume of blood required for conventional assays (0.5 to 2.5 mL per test) can lead to cumulative blood loss that equals or exceeds the circulating blood volume of an ELBW newborn [1,6]. Table 1 depicts requirements imposed by routine clinical laboratory testing when sent out from a hospital for exemplary coagulation tests [7]. The blood volume required for testing is excessive for the neonatal population, particularly when some of these tests are ordered multiple times. Reports over the past 30 years indicate that around 90% of ELBW infants and 58% of preterm infants <32 weeks of gestational age receive red blood cell transfusions and that iatrogenic phlebotomy losses and ventilatory requirements are the primary determinants for transfusion. Packed red blood cell (PRBC) transfusions may lead to a host of complications such as acute lung injury, graft-versus-host disease [2], and increased in-hospital mortality of very low birth weight (VLBW) infants [3]. Neonatologists report compromising on recommended blood draw volumes and/or omitting certain critical clinical blood tests altogether to prevent anemia [8]. Both practices can have undesired consequences including reduced testing sensitivity, higher rates of requests for repeat sample draw, or an incomplete profile of patient status if testing is omitted altogether.

While the clinical need for maximizing the diagnostic output from low blood volumes is recognized, there are only a limited number of such tests available that comprehensively address the testing needs of pediatric or newborn patients. A handful of FDA-cleared platforms have emerged as leaders in low volume testing, which include the i-STAT (Abbott Laboratories; Chicago, IL, USA), Piccolo Xpress (Abaxis Inc.; Union City, CA, USA), and Gem Premier series (Instrumentation Laboratory; Bedford, MA, USA). While these products have gained considerable traction for rapid, point-of-care blood testing of analytes important for blood chemistry and gases, cardiac care, and liver and kidney function, they only cover a subset of the ~1000 tests [7] used in routine clinical laboratory testing. Moreover, these products have a significant limitation due to their inability to perform different combinations of assays due to inherent inflexibility in the liquid handling technology. The Piccolo Xpress and Gem Premier tests utilize basic chemistry principles to measure analytes including hematocrit, blood gas, electrolytes, and glucose in blood samples. i-STAT is the only platform currently able to perform two distinct assay formats (chemistry assays and immunoassays) on the same instrument, but not the same cartridge, and there are currently no platforms capable of combining molecular analysis with other assay formats.

In this article, we describe a nanoliter scale analyzer that leverages the highly flexible liquid handling architectures inherent to digital microfluidic (DMF) technology to perform laboratory-equivalent chemistry assays, immunoassays, and molecular assays essentially on the same platform. All assays are performed in single-use, disposable cartridges that contain all assay-specific reagents onboard, unless specified otherwise. The system supports testing for a wide range of samples, including whole blood, serum, urine, saliva, and meconium (newborn stool). A novel and unique capability of DMF described here is that whole blood samples are separated into plasma on the cartridge and, as required, both whole blood and plasma samples are utilized simultaneously for various assays within the same protocol. All assays under development described here are designed specifically for vulnerable newborn and pediatric patients for whom small volume testing is especially important. Multiple assays are demonstrated utilizing microliter sample volumes dispensed from a 50 µL sample loaded into the cartridge to provide a comprehensive profile of patient status within minutes. Though each assay is performed using < one microliter of sample, choice of sample collection devices for low volumes is limited, therefore a 50 µL collection device is used.

## 2. Technology Overview

### 2.1. Digital Microfluidics Using Electrowetting

Digital microfluidics by electrowetting allows programmable manipulation of droplets (see reviews from Sista et al. 2008 [9] and Pollack et al. 2011 [10]) wherein the interfacial tension of a sub-microliter droplet is modulated on a hydrophobic surface through applied electric potential. Via the strategic application of electric potential to an array of electrodes, electrowetting facilitates simple droplet operations such as transportation across electrodes, merging to produce larger droplets, splitting to produce smaller droplets, and mixing [11,12]; or more complex droplet operations such as magnetic bead manipulation within the droplet [13], rapid thermal cycling of a droplet [14], and capture of colorimetric, fluorescence, and luminescence signals from the droplet. 

Together these features have enabled DMF by electrowetting to emerge as a highly appropriate technology for clinical testing applications, particularly where sample volume needs to be conserved while performing multiple types of assays, such as enzymatic assays [15,16,17], nucleic acid amplification (PCR) [14], immunoassays [13,18], and basic chemistry assays [19]. In an FDA-cleared product using DMF for newborn screening, enzymatic assays are performed utilizing a reaction volume of only 3 nanoliters of whole blood extracted from a dried blood spot [15]. In this article, we demonstrate the following assays each utilizing only microliter-sized sample volume per assay on the same DMF platform: (1) chemistry assays: on whole blood utilizing rate kinetic fluorescence (glucose-6-phosphate dehydrogenase; G6PD), on plasma generated within the cartridge with absorbance (albumin, total bilirubin, and unbound bilirubin), and on urine with absorbance (creatinine); (2) immunoassays: a competitive immunoassay from plasma (thyroxine; T4) and sandwich immunoassays (insulin and neutrophil gelatinase-associated lipocalin; NGAL); and (3) a molecular assay for infectious disease (cytomegalovirus; CMV). A single drop of blood is about 50 µL; assuming 1 µL sample input per assay and accounting for some dead volume, a couple dozen assays can be performed from one 50 µL blood sample.

### 2.2. Digital Microfluidic Instrument Design

**Figure 1:**
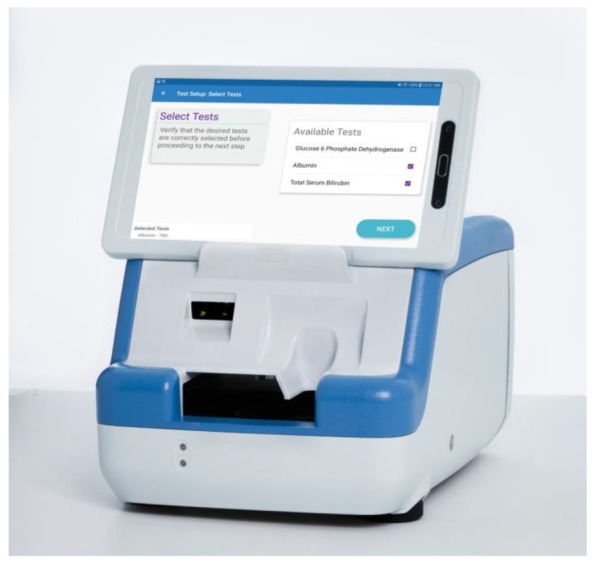
Rendering of the main components of the digital microfluidic instrument.

We developed a digital microfluidic analyzer (Figure 1) measuring 22.6 × 26.9 × 20.6 cubic centimeters and weighing less than 6.8 kg with an 8-inch mini tablet as the user interface. The mini tablet utilizes a custom designed graphical user interface (GUI) to control all droplet manipulations on the cartridge (see below). The instrument includes a cartridge deck that features motorized cartridge engagement after insertion of single-use cartridges. A second motor engages with the cartridge to release the sealed fluids (diluent containing nonionic detergent and a silicone oil filler fluid) within the cartridge for reagent preparation. The filler fluid occupies the entire chamber of the cartridge and eliminates evaporation of the liquid droplets [9]. This automated release of reagents enables the operator to perform assays without the need to introduce any external reagents into the cartridge, thereby minimizing hands-on time and operator variability. The instrument deck contains heaters that enable assays to be maintained at precise temperatures to ± 0.1 °C of the set point. The instrument contains a barcode scanner to accession patient samples and three optical detectors (400–700 nm spectrophotometer and two fluorimeters) to support a wide range of colorimetric and fluorimetric assays. All the electronics for droplet manipulation (electrowetting), thermal control, optical detection, data analysis, data transfer, and mechanical actuation are included in a single mainboard to minimize the overall footprint of the instrument. Combined with highly automated cartridge capabilities such as sample preparation and processing, the instrument has all the features required to allow its use as a walkaway system in both laboratory and near-patient settings such as the NICU or nursery.

### 2.3. Cartridge Architecture

**Figure 2:**
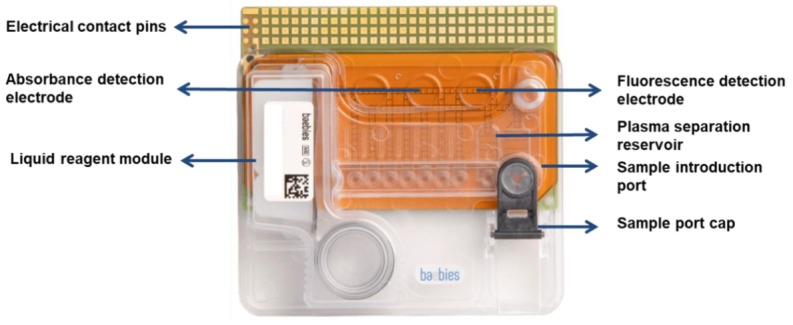
The single-use cartridge is comprised of a printed circuit board and a plastic top plate. Notable features include the sealed liquid reagent module, sample loading port, and dried reagents (not shown).

The disposable cartridge (Figure 2) is comprised of a sandwich made out of a printed circuit board (PCB) electrode “chip” and a plastic top plate with an input port for sample introduction. Droplets are manipulated within this sandwich structure. A reagent module containing liquid reagents (filler fluid and diluent) is also enclosed within the cartridge. The functional area of the cartridge is 53 mm × 81.6 mm × 0.76 mm and additional housings bring the overall envelope to 80.3 mm × 81.6 mm × 19.3 mm. All sample preparation and assay-specific reagents are dried on the PCB and automatically rehydrated on demand as required by the assay protocols. A typical assay run includes the following steps: insert the cartridge into the instrument deck, where the contact pins of the PCB (gold squares in Figure 2) automatically engage with the instrument to energize the electrodes. The cartridge is then initialized from the tablet user interface, which engages the motor to automatically release fluids into the cartridge. The user then drops a whole blood sample into the sample inlet port on the right of the cartridge and initiates the assay run protocol from the tablet user interface. All subsequent steps of the assay protocol are completely automated with no user intervention. 

A developmental version of the cartridge in which both heating and sensing electrodes are printed on the PCB itself was utilized for the molecular studies (CMV). The heaters are controlled and monitored from the instrument software and enable much faster thermal cycling than previously described aluminum heater bars, which were placed in the deck of the instrument [9]. 

### 2.4. Onboard Quality Control Checks

Numerous internal quality control (QC) checks are integrated in the system to ensure the validity of reported results. Algorithms utilize within-run optical, electrical, and thermal measurements to verify the integrity of the data generated. These algorithms can detect issues with sample and reagent loading, reagent integrity, fluidic anomalies, and plasma separation. For example, electrical impedance checks are performed on all loaded samples to ensure that sufficient sample volume is available for testing. Samples below acceptable volumes are flagged to the user as inadequate. Measurements of optical absorbance at specific wavelengths are also performed to determine whether samples have exceeded the hemolytic, lipemic, or icteric interference limits of particular assays. All QC checks are fully automated and do not require intervention or interpretation from the user.

## 3. Materials and Methods 

This section is divided into materials and methods for sample preparation, chemistry assays, immunoassays, and molecular assays with each of these sections providing further details on reagents, droplet protocols, and data analysis methods.

### 3.1. DMF Plasma Separation 

**Figure 3:**
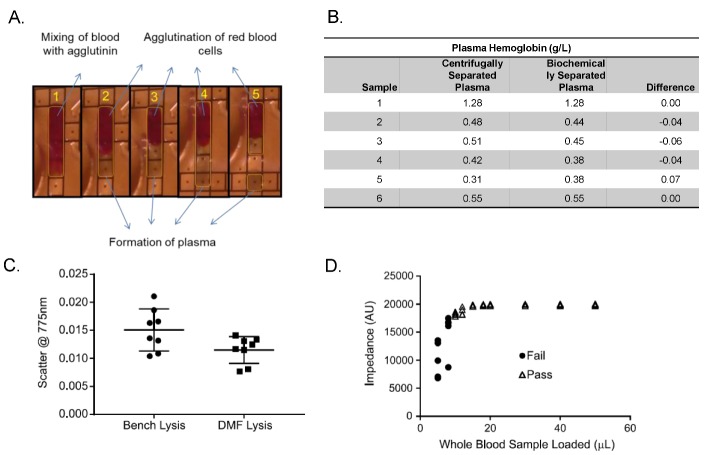
(**A**) Composite photograph of plasma separation on cartridge. (**B**) Hemoglobin levels in the plasma obtained from biochemical or centrifugation separation methods are not statistically different as measured by paired t-test. This study demonstrates that biochemical plasma separation does not induce excessive stress on the red blood cells (RBCs). (**C**) Whole blood from a single donor was lysed using either standard osmotic methods on the bench (left) or via osmotic lysis on the digital microfluidic (DMF) cartridge (right). The scatter at 775 nm is comparable in both samples, indicating that the DMF lysis method is as effective as the standard bench osmotic shock technique. (**D**) Sample input volume (x-axis) versus impedance (y-axis); circle: fail, triangle: pass. AU: arbitrary units.

A lectin-based agglutination agent was spotted directly onto the PCB and air dried. Droplets of diluent were dispensed to reconstitute the dried agglutinin and then combined with whole blood droplets dispensed from the sample input reservoir. As the whole blood mixes with agglutinin, the red blood cells (RBCs) agglutinate into a clump surrounded by liquid plasma. This plasma fraction of the mixture is manipulated by electrowetting to separate from the agglutinated cells to yield clear plasma (see Supplementary Video). Separation of plasma in this manner does not induce hemolysis (see Figure 3B). 

### 3.2. DMF Blood Lysis 

RBCs in droplets of whole blood were lysed osmotically on the cartridge by serial dilution of the blood sample with diluent. Two droplets of diluent were mixed with a single drop of blood, and then split to derive 3 droplets at a 1:3 dilution. The volume of each droplet on the cartridge is approximately 1 µL. One of the droplets is then mixed with another two droplets of diluent and this process is repeated until the necessary dilution of whole blood is obtained. Efficiency of whole blood lysis was confirmed through the absence of scatter at 775 nm.

### 3.3. Chemistry Assays

#### 3.3.1. Reagents

β-nicotinamide adenine dinucleotide phosphate (NADP+), maleimide, glucose-6-phosphate (G6P), magnesium chloride (MgCl_2_), diazonium salt, silicone oil, succinic acid, glucose, glucose oxidase, peroxidase-HRP, bilirubin, dyphylline, excipients, surfactants, preservatives and stabilizers were procured from MilliporeSigma (St. Louis, MO, USA). Bromocresol Green (BCG) was obtained from Affymetrix (Santa Clara, CA, USA). Creatinine kits (2-step colorimetric readout) were purchased from Abcam (Cambridge, UK). Urine calibrator samples were procured from CST Technologies (Great Neck, NY, USA). 

#### 3.3.2. Droplet Manipulation Protocols

The G6PD, total bilirubin (TBil), and albumin assays were performed using reagents that were spotted on the PCB and air dried under controlled temperature and humidity.

For determination of G6PD activity, red blood cells were first lysed osmotically in the cartridge by mixing whole blood with diluent as described above. The lysed sample droplet was then mixed with assay-specific reagent 1 (5 mM NADP+, 20 mM maleimide, and excipients in 20 mM phosphate buffer, pH 6.3). Addition of a second reagent (2 mM G6P, 5 mM NADP+, 2.4 mM MgCl_2_, and other additives in 100 mM phosphate buffer, pH 7.3) induced production of NADPH. Kinetic fluorescence measurements were collected by shuttling the reaction droplet around a loop of electrodes where every 10 s the fluorescence is collected at the designated detection electrode for a period of approximately 4 min. The rate of NADPH production is then quantified from the increasing fluorescence, which is proportional to G6PD enzymatic activity. The reaction occurs in the presence of maleimide, which is used to improve the specificity of the assay by minimizing the production of NADPH from 6-phosphogluconate dehydrogenase. Hemoglobin present in the lysed droplet was measured through spectral absorbance and used to normalize G6PD enzymatic activity, resulting in a final reported value of units activity per gram hemoglobin (U/gHb). 

TBil and albumin were measured using azobilirubin and bromocresol green (BCG) chemistries, respectively. The previously described biochemical technique for on-cartridge plasma separation was performed prior to each assay. To measure TBil, a droplet of the separated plasma was combined with dried reagent (2 mM diazonium salt, 2.0 mM dyphylline, 150 mM NaCl, and excipients in 50 mM maleate buffer, pH 3.2). It should be noted that plasma is directly used to reconstitute the dried reagent. Bilirubin in the plasma transforms into azobilirubin, which caused a proportional increase in absorbance. To measure albumin, a droplet of the separated plasma was combined with a droplet of 1.0 mM BCG, surfactants, and excipients in 100 mM succinate buffer (pH 3.6) to form a complex with albumin, the concentration of which was proportional to the absorbance intensity. 

Unbound bilirubin (UB) was measured using liquid reagents according to the protocol by Shimabuku and Nakamura [20] in which glucose and glucose oxidase are present in significant excess to turn over glucose with H_2_O_2_ as one of the end products. A single droplet of bilirubin calibrant was combined with a droplet of the reaction mixture and transported to the detector, where the spectrophotometric characteristics of the un-oxidized bilirubin were followed over time to detect the time-dependent pattern of oxidation. The amount of unbound bilirubin is then correlated to the rate of oxidation. 

The creatinine assay was performed using liquid reagents as follows. Undiluted urine calibrant samples or clinical samples were dispensed from the sample reservoir; a single droplet of sample was then combined with the reaction mix and incubated. The reaction droplet was then transported to the detection electrode where absorbance was measured. 

### 3.4. Immunoassays

#### 3.4.1. Reagents

Superparamagnetic bead immunoassay kits for free thyroxine (T4) and insulin were procured from Beckman Coulter (Brea, CA, USA). Superparamagnetic beads for the neutrophil gelatinase-associated lipocalin (NGAL) immunoassay were conjugated in-house using Dynal MyOne goat anti-mouse beads (ThermoFisher, Waltham, MA, USA) with NGAL capture and detection antibodies obtained from R&D Systems (Minneapolis, MN, USA) and ThermoFisher. 4-methylumbelliferyl phosphate (4MUP) was procured from MilliporeSigma.

#### 3.4.2. Droplet-Based Magnetic Bead Immunoassay Protocol

All immunoassays were performed using liquid reagents, which were loaded into the cartridge prior to initiation of the automated immunoassay protocol. 

For the free T4 assay (a competitive immunoassay), one droplet (approximately 1 µL) of sample was combined with a droplet containing the T4 capture antibody (conjugated to magnetic beads), and an excess of biotinylated T4 analog. Endogenous T4 in the sample competes with the biotinylated T4 and the fluorescence output of the assay from 4-MU derived from biotin-bound-streptavidin ALP is inversely related to the T4 level. The insulin and NGAL assays were sandwich immunoassays in which capture and detection antibodies form a “sandwich” with the analyte of interest and the final fluorescence signal generated is directly proportional to the amount of analyte. With some minor modifications, protocols described in Sista et al. [13] were followed. Briefly, one droplet of sample was mixed and incubated with antibody conjugated magnetic beads within the cartridge. The droplets were then transported to a high-density magnetic gradient where the magnetic beads were washed. Finally, a fluorescence substrate (4MUP) is added to the washed beads and transported to the detector, where the fluorescence signal was measured kinetically at 360 nm excitation/460 nm emission. The rate of increase in fluorescence was used to calculate the concentration of the analyte using a standard curve generated on the cartridge.

### 3.5. Molecular Assays

#### 3.5.1. Reagents

The real-time PCR mixture and polymerase were procured from Roche Custom Biotech (Indianapolis, IN, USA), primers and probe (6-carboxyfluoescein; 6-FAM) were obtained from Applied Biosystems (Foster City, CA, USA) and ThermoFisher using the CMV sequences published by Boppana et al. [21], and custom sequences for lambda and probe labelled with Texas Red were obtained from Integrated DNA Technologies (Coralville, IA, USA). Calibrants were generated by diluting purified CMV (National Institute of Standards and Technology; Gaithersburg, MD, USA) or lambda phage control DNA (ThermoFisher) with water and spiking it into the master mix at concentrations ranging from 1 to 10 copies/μL. The CMV target for analysis was the immediate early exon 5 region with forward primer 5′-GAGCCCGACTTTACCATGCA-3′, reverse primer 5′-CAGCCGGCGGTATCGA-3′ and probe 5′-ACCGCAACAAGATT-3′. The lambda DNA forward primer was 5′-CATCAAAGCCATGAACAAAGCAGC-3′, Reverse primer 5′-GCCGCAGCCTGTTAACCTG-3′, and probe 5′-GGATGAACTGATACCGGGGTTGCTGAGTG-3′. 

#### 3.5.2. On-Cartridge DNA Amplification Protocol

Target DNA sequences were amplified on the cartridge using heaters printed onto the PCB itself, which are rapidly thermocycled using the instrument software. Fluorescence output from amplification of the 6-FAM probe was captured using the instrument fluorimeter. Reaction droplets (each containing sample, master mix, primers, probes, and reference DNA) were subjected to 50 cycles, with each cycle consisting of a 2-s denaturing stage (93 °C), followed by a 2-s extension stage (60 °C). The fluorescence output of each cycle was detected at the site of amplification such that the entire 50 cycle reaction, including on-cartridge operations such as droplet dispensing, reagent mixing, and reaction setup, was completed in 5 min. Note that nucleic acid extraction from more robust organisms or matrices (e.g., fungi, bacteria, blood, stool, etc.) may require additional steps, which have been demonstrated on the DMF platform [9]. 

### 3.6. Acquisition of Clinical Samples

Samples utilized for the method comparison studies were from adults, obtained from the following sources: G6PD clinical samples were purchased from BioIVT (Westbury, NY, USA) as whole blood samples. Plasma samples for albumin and bilirubin were purchased from Discovery Life Sciences (Huntsville, AL, USA). Procurement of samples from neonates or pediatric patients was not feasible as blood collection volumes needed for comparator assays were often milliliters of blood.

Serum samples utilized for insulin and free T4 studies were assayed adult plasma samples (discarded and de-identified but with associated clinical values) obtained by collaborators at Duke University under an IRB approved protocol. Samples were stored at −80 °C for 3 to 36 months prior to testing. 

Assayed urine samples utilized for the NGAL and creatinine studies were obtained from the University of Alabama at Birmingham under IRB approved protocols (discarded and de-identified). 

### 3.7. Reference Methods for Method Comparison Studies

For each of the method comparison studies, the DMF assays were compared to a currently validated assay as outlined in the following. 

For the albumin and TBil assays, human serum and heparinized plasma samples were procured from a research sample biobank (Discovery Life Sciences, Huntsville, AL, USA) and added to washed human red blood cells (BioIVT, Westbury, NY, USA) to target 50% hematocrit. Each whole blood sample was analyzed using three different lots of cartridges. The remaining prepared whole blood sample was centrifuged to obtain plasma, which was shipped to LabCorp (Burlington, NC, USA) for measurement (Hepatic Function Panel) using standard laboratory equipment used for routine clinical reporting (Roche Cobas analyzer). Albumin and total bilirubin values obtained from LabCorp were used as comparator values. For the G6PD assay, heparinized adult whole blood (BioIVT) was obtained and analyzed using three different lots of cartridges. The remaining whole blood was sent to LabCorp for G6PD measurement using the Pointe Scientific reagent kit on the Roche Cobas analyzer. All biological material for the albumin, TBil, and G6DP method comparison studies were derived from adult donors, with no specification on gender, ethnicity, or age range.

For the unbound bilirubin assay, human plasma samples that were obtained from Discovery Life Sciences values were acquired using an in vitro diagnostic clinical analyzer. Creatinine values were acquired using a mass spectrometry based method run in the University of Alabama at Birmingham clinical laboratory. NGAL values were acquired using a Kidney Injury Panel 5 Human kit (KIP-5) microtiter plate assay (Meso Scale Discovery, Rockville, MD, USA) [22]. Insulin levels were measured using the ACCESS ultrasensitive insulin assay kit from Beckman Coulter (Brea, CA, USA). Free T4 values were measured using the Access Free T4 Beckman Coulter Kit. 

## 4. Results

### 4.1. Integration of Sample Pre-Analytical Steps

Here, we describe integration of some of the pre-analytical steps such as plasma preparation, red blood cell lysis, and sample quality assurance, which are all required sample preparation and processing steps to deliver the analyte to the analytical assay.

#### 4.1.1. Plasma Preparation

Due to the inherent simplicity in use of DMF technology and flexibility in its operation, the platform is designed for use in multiple settings including a hospital laboratory, NICU, physician’s office, or other distributed settings without ancillary laboratory equipment. We developed integrated protocols for common sample preparation requirements including plasma separation from whole blood, RBC lysis, and DNA isolation on the same cartridge. For plasma separation, we utilized a biochemical method which is based on lectin-induced agglutination to facilitate clumping of the RBCs directly on the cartridge [23]. As illustrated in Figure 3A, agglutinated red cells are no longer responsive to electrowetting, while the supernatant plasma droplets from the agglutinated sample can be transported away from the agglutinated red blood cells. Matched pairs of samples were also subject to centrifugal separation (at 1200 RCF for 2 min) to obtain plasma. Hemoglobin levels are then measured optically using its extinction coefficient. It was determined that there was no significant increase in the hemoglobin levels of cartridge separated plasma samples compared with centrifuged plasma samples (Figure 3B). 

#### 4.1.2. Red Blood Cell Lysis

We have also demonstrated the capability to perform red blood cell lysis from whole blood on the DMF cartridge. RBC lysis is performed using the osmotic method which incorporates serial dilution of the blood sample with water. To demonstrate the efficacy of on-board DMF hemolysate preparation, whole blood from a single donor was serially diluted within the cartridge and the optical scatter was measured at 775 nm, providing a surrogate measure of incompletely lysed red blood cells (Figure 3C). The same whole blood sample was also subjected to the standard hemolysate preparation method [24] and then loaded into the cartridge for comparison. We found no significant differences in scatter at 775 nm, suggesting that the DMF red blood cell lysis protocol is as effective as the standard hemolysate preparation procedure.

#### 4.1.3. Sample Volume Check

The cartridge can accept a wide range in input sample volume variations from the sample transfer pipette. Figure 3D depicts impedance measurements in loaded whole blood sample droplets ranging from 5 to 50 μL. Quality control impedance checks are performed on all samples during the automated assay protocol to ensure that sufficient sample volume is available for testing. The use of impedance to interrogate sample volumes relies on the dielectric property of filler fluid which confers high electrical resistivity to all electrodes in the cartridge. The presence of aqueous droplets on the electrodes displaces filler fluid and changes the impedance in the circuit in a volume dependent manner. Samples below 10 μL are flagged as inadequate (fail) and shown with the circle symbols. Within this acceptable input sample volume range of 10–50 μL, the precision for dispensed test volume droplets is high as it is derived from the lithographically defined electrodes used for dispensing droplets with electrowetting. 

### 4.2. Miniaturized, Automated Diagnostic Assays

Our DMF platform is the first of its kind to combine chemistry assays, immunoassays, and molecular assays together on a single system. Over three dozen unique assays relevant for neonatal diagnostics utilizing multiple types of samples have been demonstrated on our DMF platform so far and some are summarized in Table 2. The maturity of these assays varies, spanning from early stage feasibility demonstration to advanced stages of product development. In the following sections, we present representative assays for each of the major classes shown in Table 2. 

#### 4.2.1. Chemistry Assays

**Figure 4:**
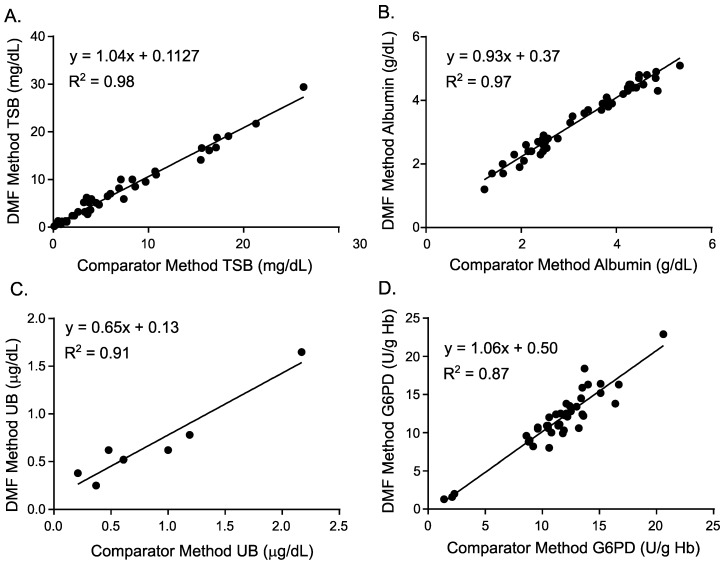
Method comparison results of the DMF assays (y-axis) to comparator laboratory assays performed at LabCorp (x-axis) for total bilirubin (TBil) (**A**), albumin (**B**), and glucose-6-phosphate dehydrogenase (G6PD) (**D**), or using an in vitro diagnostic comparator method for unbound bilirubin (**C**).

Among the most ordered chemistry assays for neonates are TBil and albumin, which inform neonatal hyperbilirubinemia diagnosis and subsequent treatment based on American Academy of Pediatrics guidelines for hyperbilirubinemia [25]. Standard dye-binding chemistries based on diazo and BCG dyes for TBil and albumin, respectively, were adapted to the DMF platform by developing droplet movement protocols that coordinate step-wise plasma separation, rehydration of the dried reagents for each assay, merging of sample droplets with reagents, and detection. Method comparison studies of the DMF TBil and albumin assays to comparator methods on Roche Cobas are shown in Figure 4A,B, respectively, and demonstrate strong correlation.

Whereas TBil measurement is the current standard of care in hyperbilirubinemia assessment, it primarily recognizes bilirubin in its bound form and does not accurately measure unbound bilirubin —the form of bilirubin that is able to cross the blood-brain barrier and has been implicated in kernicterus and other neurological sequelae of severe hyperbilirubinemia [26]. Unbound bilirubin is regarded as the most accurate biomarker for risk of bilirubin-induced neurological dysfunction (BIND) and kernicterus, but is not widely used as current assays are technically challenging with several manual sample processing steps. We developed a DMF protocol for unbound bilirubin measurement, which is based on the method developed by Jacobsen and Wennberg [27] and later modified by Ahlfors [28,29]. Briefly, the method relies on Michaelis–Menten enzyme rate kinetics of the oxidation of serum bilirubin to derive the concentration of free bilirubin. Bound bilirubin is protected from oxidation and only unbound bilirubin (B_free_) is oxidized and detected kinetically according to the rate of decreasing absorbance. The DMF unbound bilirubin assay successfully measures the loss of absorbance in calibrant samples and results of a preliminary method comparison study (Figure 4C) indicate promising correlation of the DMF assay with a standard reference method. These results demonstrate the proof of concept for a fluidically complicated assay (with many fluidic operations) that is ideally suited to exploit the sophisticated fluid control available on a digital microfluidics platform. Future work will incorporate additional standardization steps to the protocol and optimize reagent storage on the cartridge to facilitate complete automation of the assay. 

Another important assay that is used to identify the risk factors for neonatal hyperbilirubinemia is glucose-6-phosphate dehydrogenase (G6PD) enzyme activity, which identifies the relatively common deficiency of this enzyme (1 in 8 African American males are affected [30]). Patients with G6PD deficiency experience hemolysis in their circulation, causing elevated levels of bilirubin in their bloodstream. Screening all newborns for G6PD deficiency status prior to hospital discharge has been advocated, but is not yet implemented due in part to the lack of acceptable, rapid tests [31]. 

Here, we describe the development of a fully automated quantitative sample-to-answer DMF G6PD enzyme assay from 50 μL of whole blood. Onboard controls ensure that the operational temperature of the cartridge is regulated tightly, an important feature given that G6PD activity is highly dependent on temperature fluctuations. An automated osmotic shock lysis procedure (shown in Figure 3) was also designed to release the G6PD enzyme from red blood cells. Finally, automated absorbance measurements were used to determine hemoglobin levels that are needed to normalize G6PD activity between patients. It should particularly be noted that assays for TBil, albumin, and G6PD were all performed on the same cartridge, with each assay requiring only 1–2 μL of whole blood. The detection method was absorbance for TBil and albumin, while rate kinetic fluorescence measurements were utilized for G6PD. Method comparison studies of the DMF G6PD assay to a comparator method on Roche Cobas are shown in Figure 4D and indicate strong correlation. 

**Figure 5:**
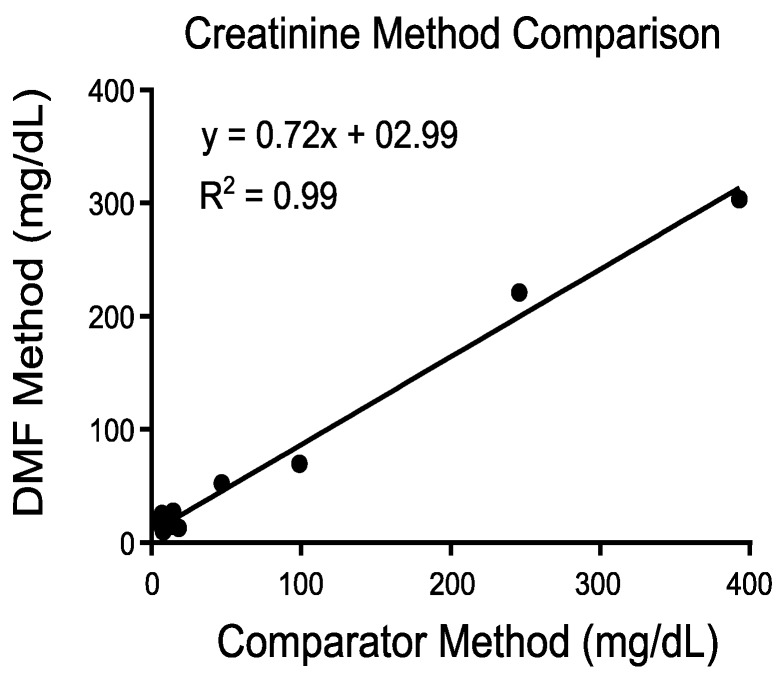
Analysis of urine samples using DMF. Method comparison using the DMF method (y-axis) for creatinine versus the comparator method using mass spectrometry (x-axis).

While low blood volumes are generally preferred in newborn and pediatric patients for the stated reasons, minimizing the sample volume for other physiological samples, such as urine, is also highly desirable in this population. To broaden the compatibility of different types of samples, we have demonstrated a DMF assay for creatinine, which is commonly tested in urine (Figure 5). Urine generally requires less sample preparation than blood samples. Samples can be loaded neatly onto the DMF cartridge and diluted on the cartridge using the automated assay protocol. Following sample dilution, the assay proceeds as previously described for blood chemistry assays: a droplet of sample is combined with a droplet of reagent, mixed, and transported to the detector for analysis. Method comparison of the DMF creatinine assay is shown in Figure 5 covering the clinically relevant range and indicates strong correlation of the DMF assay to a comparator assay. 

#### 4.2.2. Immunoassays

Immunoassays are another commonly used method in clinical laboratory testing to measure antibodies, antigens (proteins, hormones, small molecules, etc.), or a combination of the two. We previously published DMF immunoassay protocols for Troponin I, insulin, and IL-6 [9,13] using antibody conjugated magnetic beads and chemiluminescence detection. Mechanisms for microfluidic manipulation of magnetic beads, capture of beads with a stationary magnet, and efficient bead washing were rigorously characterized using a previous cartridge design [9]. We developed several immunoassays using the published protocol [13] on the current smaller DMF cartridge with the notable, albeit minor, modification to using fluorescence as the mode of detection. Briefly, the droplet-based immunoassay procedure includes: (1) a sample droplet is combined with a droplet containing magnetic beads conjugated to primary capture antibodies; (2) the combined droplet is then merged with a third droplet containing the secondary antibody labeled with alkaline phosphatase (ALP; reporter) and incubated for the desired period of time to enable formation of antibody-antigen-reporter complex; (3) the magnetic beads are then immobilized with a magnet and washed several times to remove unbound material; (4) fluorescence substrate is merged with the washed bead droplet, resulting in the production of fluorescence from the enzyme-substrate reaction; (5) the droplet containing the final reaction is transported to the detection electrode to capture fluorescence output. All samples analyzed in this section utilized plasma as the sample input in order to illustrate the flexibility of the platform. Future steps will incorporate automated plasma separation to enable whole blood input for immunoassays. 

**Figure 6:**
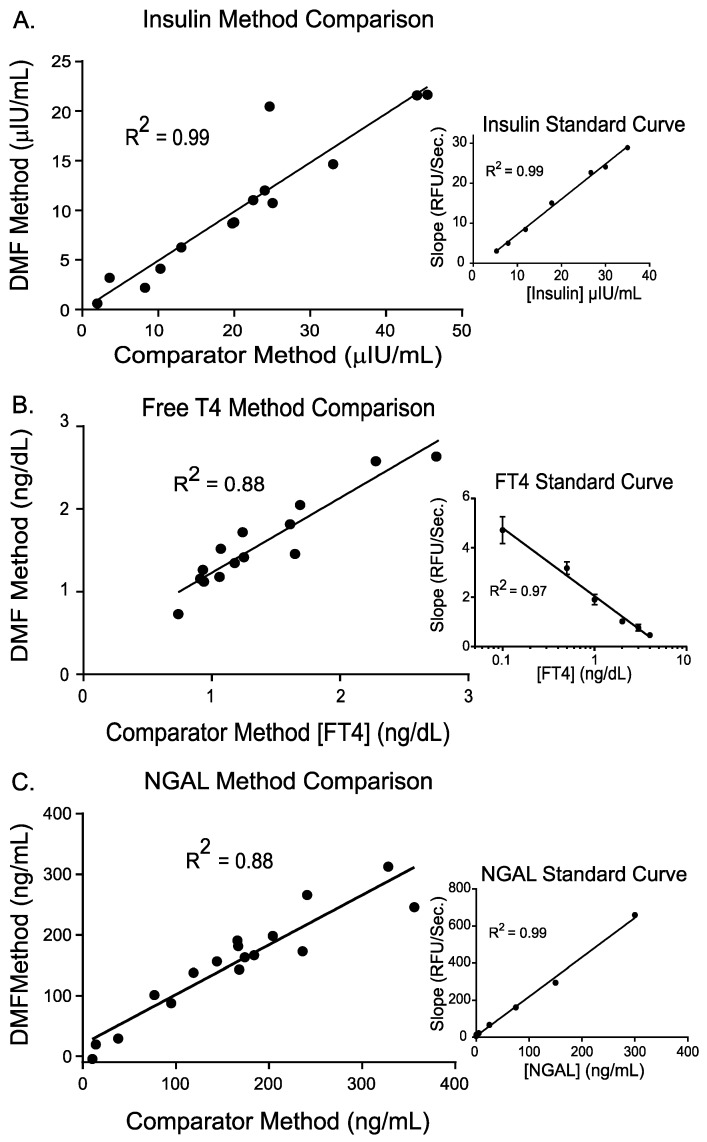
Immunoassay results for insulin (**A**), free T4 (**B**) and neutrophil gelatinase-associated lipocalin (NGAL) (**C**) on the DMF cartridge. (**A**,**B**) are sandwich and competitive immunoassays, respectively, using plasma calibrant samples; (**C**) is a sandwich immunoassay using urine calibrant samples.

Insulin assays are routinely ordered for newborns and children suffering from persistent hypoglycemia and access to quick results, while still minimizing blood volume, is desirable under such conditions. As shown in Figure 6A, the resulting insulin sandwich immunoassay was linear across the desired range for newborns and pediatric patients, and the DMF method compares well against a reference method. Results from preliminary work (data not shown) indicate that 50 µL of whole blood would be sufficient to perform a full hypoglycemia panel consisting of insulin, cortisol, growth hormone, glucose, beta hydroxybutyrate, and free fatty acids as the sample utilized for each assay is about 1 µL. 

Free thyroxine (T4) is one of the most commonly ordered tests for newborns to investigate potential congenital thyroid disorders such as neonatal Graves’ disease and congenital hypothyroidism (incidence of 1 in 3000 births). We have demonstrated a competitive immunoassay for free T4 on the DMF cartridge. Competitive immunoassays are often used for small molecules and are based on the competition of the native analyte with an alkaline phosphatase (ALP) labeled version of the analyte; the two analytes compete for binding by a primary capture antibody. Addition of 4MUP induces signal generation from the ALP-labeled FT4 analogue and the signal produced is inversely proportional to the amount of analyte in the sample. Results of our DMF competitive immunoassay for free T4 are shown in Figure 6B. The assay is linear across the clinical range for newborns and pediatric patients and method comparison studies against a reference method showed good correlation. 

While many clinical immunoassays are performed on blood samples, other biological samples may be tested to discern various etiologies. Kidney function is a common concern for preterm newborns that often requires analysis of urine samples. We also demonstrated an immunoassay for neutrophil gelatinase-associated lipocalin (NGAL) on urine samples on the DMF cartridge (shown in Figure 6C). The DMF NGAL assay utilized a droplet manipulation program that was very similar to that described for insulin. The resulting assay was linear across the desired range for newborns and pediatric patients, and the DMF method compares well against a reference method. 

#### 4.2.3. Molecular

Molecular diagnostic laboratory tests are commonly used for the detection of infectious disease and genetic markers of disease risk. Recently published DMF protocols for polymerase chain reaction (PCR) from human genomic DNA, bacterial genomic DNA, and fungal genomic DNA [9,10] used methods in which the heaters were embedded in the cartridge deck and the droplets were shuttled between two or more thermal zones or thermal cycled in place with a block heater to amplify molecular targets. In order to decrease PCR cycle times, we embedded heating and sensing elements into the diagnostic cartridge to reduce the thermal mass and localize thermal cycling to a single electrode with the 1 µL droplet. The reduced thermal mass led to shorter cycle times and obviated the need for a cooling fan. Moreover, such capability will also enable a battery-operated device as the power consumption is significantly reduced. The heaters are controlled and monitored from the instrument software and support exceptionally fast cycling conditions (3–4 s per cycle). 

**Figure 7:**
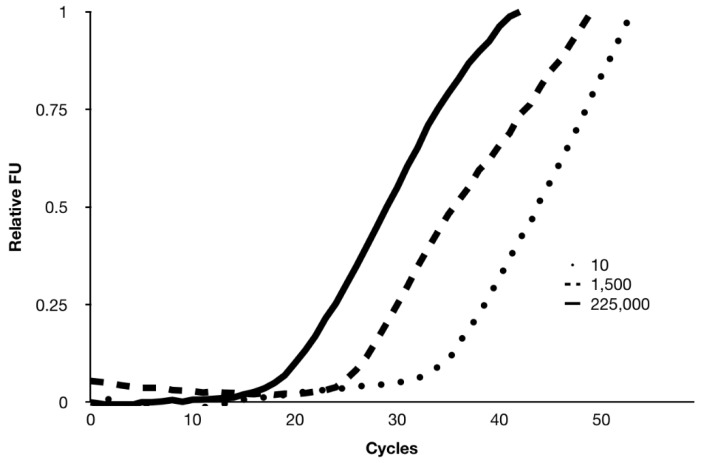
Cytomegalovirus (CMV) polymerase chain reaction (PCR) assay using a prototype DMF cartridge. Successful amplification of CMV (10, 1500, and 225,000 copies/µL) is achieved within 5 min. FU: fluorescence units.

Congenital CMV (cCMV) infection increases a child’s risk for long-term health problems including hearing loss, and newborn screening for cCMV has been advocated [32]. The mean CMV DNA copy number in newborns with cCMV was 1790 copies/µL and it was much higher in those newborns that had both sensorineural hearing loss and cCMV [33]. Using a prior cartridge and instrument-based heater design, we have shown feasibility of CMV amplification in a DMF system using saliva samples [34]. Figure 7 shows that in the novel cartridge design with built-in heaters and sensors is capable of amplifying CMV at concentrations of 10, 1500, and 225,000 copies/µL. The C_t_ values were 32, 26, and 19 respectively for 10, 1500, and 225,000 copies/µL, which is generally in line with expected relative cycle threshold values. An internal control lambda (600 copies/µL) was also multiplexed along with CMV (data not shown). PCR amplification completed within 5 min. These results show promise for performing rapid PCR with further optimization of thermal cycling of on-board heaters, reagent composition, and the reaction conditions.

## 5. Conclusions

The DMF platform described in this report is the realization of a technology that was envisioned over 10 years ago [9]: a small footprint, automated liquid handling system that reduces sample volume requirements for common clinical tests across testing methodologies and types of samples with a short turnaround time. Owing to the limitations in the flexibility and sophistication of microfluidics employed, many established point-of-care testing devices are only able to handle a limited array of assay methodologies in the same cartridge. Though research groups have shown such capabilities, to date there is no commercially available platform with such flexibility in customization of assay panels. Our DMF platform combines three disparate assay types (chemistry assays, immunoassays, and molecular assays) in a single, near-patient device utilizing the same underlying droplet manipulation protocols on a cartridge. This unique capability expands the clinical reach of our assays beyond typical chemistry assays or immunoassays to include molecular diagnostic testing such that custom panels of tests encompassing all these methodologies can be developed. Novel features such as dried reagents and integrated sample preparation on the cartridge not only reduce the hands-on effort, but also reduce potential error associated with reagent handling. These capabilities further reduce the costs for labor. 

While quality control checks are routinely implemented in mature technologies, a number of measurements of optical, electrical, and thermal parameters have been uniquely developed for the droplet handling platform. Several algorithms have been developed to flag any issues with sample loading, reagent integrity, fluidic anomalies, and blood separation—all of which are novel and essential to mature the DMF platform to produce diagnostic quality results. Chemistry assays including sample preparation of whole blood (removal of red blood cells and cell lysis) were demonstrated on a DMF cartridge spanning dye-binding colorimetric assays (albumin and total bilirubin) to rate kinetic fluorimetric enzymatic assays (G6PD) with dried reagents onboard in around 15 min including the sample preparation pre-analytical steps. Another chemistry assay (creatinine) with urine samples has also been demonstrated, as well as feasibility of an unbound bilirubin assay from plasma. Utilizing the same DMF platform, representative data are shown for competitive immunoassays (free T4) and heterogeneous sandwich immunoassays (insulin and NGAL) from plasma and urine samples, respectively. Additionally, by integrating the heaters onto the same DMF cartridge, a rapid PCR assay (CMV) has been demonstrated within 5 min. Precise droplet control in a DMF cartridge, coupled with the novel features presented, enable results that are comparable to standard clinical laboratory assays, but using only 1 µL sample volume and with greater flexibility. Interference and other analytical validations of the DMF assays are outside of the scope of this publication, however this information will be included in follow-up publications. 

The DMF platform described in this report is demonstrated to possess all the features that are required to bring the advantages of low sample volume and rapid testing to neonatal and pediatric diagnostics. 

## Figures and Tables

**Table 1:** Requirements for routine coagulation testing.

Analyte	Reference Method [7]	Expected Turnaround Time	Minimum Volume of Plasma/Serum	Estimated Volume of Whole Blood	Additional Pre-Shipping Sample Preparation
Heparin Anti-Xa	Labcorp 117101 (activity assay)	1–3 days	1.0 mL	2.0 mL	Centrifugation right after collection and frozen shipping
Factor VIII activity	Labcorp 500192 (activity assay)	3–5 days	0.5 mL	1.0 mL	Same as above
ATIII activity	Labcorp 015040 (activity assay)	2–3 days	1.0 mL	2.0 mL	Same as above
Von Willebrand factor antigen	Labcorp 086280 (immunoassay)	1–3 days	1.0 mL	2.0 mL	Same as above
Protein S antigen	Labcorp 164517 (immunoassay)	2–3 days	2.0 mL	4.0 mL	Same as above
Factor V Leiden Mutation Analysis	Labcorp 511154 (nucleic acid assay)	5–7 days	n/a	3.0 mL	None
Factor II (Prothrombin), DNA Analysis	Labcorp 511162 (nucleic acid assay)	5–7 days	n/a	3.0 mL	None

**Table 2:** Summary of assays adapted to the diagnostic DMF platform.

Sample Matrix	Immunoassays		Molecular		Chemical/Enzymatic Assays	
	Competitive	Sandwich	Genomic (Eukaryotic)	Infectious Disease (Prokaryotic)	Functional	Biochemical
Blood	Free T4, cortisol	Insulin, human growth hormone, protein S, von Willebrand factor, thyroid stimulating hormone, creatine kinase MM	Factor II mutation, factor V Leiden	HIV	G6PD, antithrombin III, protein C, factor VIII, galactose-1-phosphate uridyltransferase	Total bilirubin, albumin, unbound bilirubin, glucose, beta-hydroxybutyrate, free fatty acids, factor Xa, phenylalanine, creatine kinase, glutamine, glutamate, ammonia
Urine	Fentanyl	NGAL, Cystatin C				Creatinine
Saliva				CMV		

## Supplementary Materials

The following are available online at https://www.mdpi.com/2075-4418/10/1/21/s1, Video S1: Automated DMF protocol for G6PD, TBil, and albumin measurement from whole blood.
