# Supplementary material for: Digital Microfluidic Platform to Maximize Diagnostic Tests with Low Sample Volumes from Newborns and Pediatric Patients
Source: Diagnostics (Basel). 2020 Jan 1;10(1):21. doi: 10.3390/diagnostics10010021 (PMC7169462; doi:10.3390/diagnostics10010021)
Supplement: Supplementary file 1 [file diagnostics-10-00021-s001.zip › diagnostics-679498 supplementary/diagnostics-679498-video caption.rtf]

Automated DMF protocol for G6PD, TBil, and albumin measurement from whole blood. Video is accelerated to ~15x normal speed. The liquid reagent (left reservoir) and sample (right reservoir) are artificially dyed to enhance visibility. Droplet operations including liquid dispense, reconstitution of dried reagents, red blood cell lysis, plasma separation from whole blood, sample dilution, fluorescence measurements, and absorbance measurements are labeled at appropriate times during the video.
